# OCTA reveals remodeling of the peripheral capillary free zones in normal aging

**DOI:** 10.1038/s41598-021-95230-0

**Published:** 2021-08-02

**Authors:** Edmund Arthur, Jessica Alber, Louisa I. Thompson, Stuart Sinoff, Peter J. Snyder

**Affiliations:** 1 Department of Biomedical and Pharmaceutical Sciences, University of Rhode Island, Kingston, RI USA; 2 George and Anne Ryan Institute for Neuroscience, University of Rhode Island, 130 Flagg Road, Kingston, RI 02881 USA; 3 Butler Hospital Memory and Aging Program, Providence, RI USA; 4 Department of Psychiatry and Human Behavior, Alpert Medical School of Brown University, Providence, RI USA; 5 BayCare Health, Clearwater, FL USA

**Keywords:** Biomarkers, Medical research

## Abstract

The retinal neurovascular unit consists of blood vessel endothelial cells, pericytes, neurons, astrocytes, and Müller cells that form the inner retinal blood barrier. A peripheral capillary free zone (pCFZ) represents the distance that oxygen and nutrients must diffuse to reach the neural retina, and serves as a metric of retinal tissue oxygenation. The pCFZs are formed based on oxygen saturation in the retinal arterioles and venules. Because retinal arterioles contain a larger concentration of oxygenated blood than venules, there is a reduced need for capillaries to exist closely to arterioles compared to venules. Therefore, in a healthy individual, larger periarteriole CFZs are expected compared to perivenule CFZs. With normal aging, there is atrophy of the inner retinal neurons, and consequently reduced extraction of oxygen and nutrients from the retinal vessels (i.e., increased oxygen saturation). Therefore, we hypothesized that the peripheral CFZ will remodel with normal aging. Using Optical Coherence Tomography Angiography, we showed that the pCFZs do remodel in normal aging with large (perivenule: η^2^_p_ = 0.56) and moderate (periarteriole: η^2^_p_ = 0.12) effect sizes, opening the possibility that such changes may be further increased by neurodegenerative diseases that adversely impact the health of the retinal neural cell layers.

## Introduction

Aging is the strongest risk factor for many neurodegenerative diseases^1^. While it is hypothesized that there is an increase in the area of the foveal avascular zone (FAZ) in normal aging^2–5^, some studies have shown no difference in the FAZ area in younger and older adults^6–8^. There is also atrophy of the inner retinal neurons; specifically, the retinal nerve fiber layer (RNFL) and retinal ganglion cell (RGC) layer in normal aging^9–11^. The atrophy of the RNFL and RGC layer consequently lead to reduced oxygen extraction and a paradoxical increased oxygen saturation in the arterioles and venules in the retina, which has been measured using traditional retinal oximetry methods^12,13^. Optical Coherence Tomography Angiography (OCTA) has shown reduced vessel density in older adults compared to younger adults^4,14^.

Larger FAZ area has been shown in the retina of older adults compared to younger adults using Fluorescein Angiography (FA)^2,3^ and OCTA^4,5^. However, there is large individual variability in the size of the FAZ (co-efficient of variation ~ 50%)^15,16^, which may account for disparate results in studies examining the FAZ area in normal aging^6–8^. In studies showing larger FAZ areas in cognitively unimpaired older adults, individual FAZ values in the cognitively unimpaired older group may not exist outside the one-tailed 95% confidence interval of the younger group, thus limiting the sensitivity of the use of the FAZ area as a metric to detect subtle retinal vascular changes in normal aging^17^. Traditional retinal oximetry methods are highly variable^18,19^, and vessel density computation in OCTA images can be affected by noise in the image along with variable anatomical features such as vessel diameter^20^. Hence, finding a biomarker that is not limited to the fovea, and less likely to be affected by noise in the image, as well as a distance metric that is less variable compared to traditional retinal oximetry methods, may prove useful in detecting subtle peripheral retinal changes in normal aging.

We have previously characterized and quantified metrics of retinal tissue oxygenation termed the peripheral capillary free zones (pCFZs) in cognitively unimpaired young adults using two non-invasive retinal imaging modalities; OCTA and Adaptive Optics Scanning Laser Ophthalmoscope (AOSLO)^17^. We quantified and characterized two types of pCFZs; the periarteriole CFZ (defined as the linear distance from an arteriole to the middle of the nearest capillary) and the perivenule CFZ (defined as the linear distance from a venule to the middle of the nearest capillary)^17^. Periarteriole CFZs develop as a result of vaso-inhibitory effects of oxygen diffusing out of the retinal arterioles^21^. The higher oxygen concentration in the retinal arterioles results in reduced need for retinal capillaries to exist closely to the arterioles to supply the retinal neurons, and hence large periarteriole CFZs^21–25^. The retinal venules contain a lower concentration of oxygenated blood, creating an increased need for retinal capillaries to exist closely to the venules to supply the retinal neurons, and therefore narrower pCFZs exist around the venules (perivenule CFZs) than the arterioles^17,23,25^. We found the periarteriole CFZ to be significantly larger than the perivenule CFZ^17^. The perivenule CFZ was also found to be significantly positively associated with vessel distance from the fovea, and vessel diameter indicating consistency of the perivenule CFZ^17^. We also provided confidence limits for the pCFZs in cognitively unimpaired young adults (22–30 years)^17^.

The loss of retinal vessel density^4,14^ and atrophy of the inner retinal neurons^9–11^, concomitant with reduced oxygen extraction (increased oxygen saturation) from the arterioles and venules in normal aging^12,13^ suggests the possibility of increasing size of the pCFZ width in normal aging. The pCFZ is less likely to be affected by signal noise compared to the computation of vessel density in OCTA images because these dark areas around arterioles and venules on OCTA are readily discernible^17^. The pCFZ is not limited to the fovea and therefore can detect more subtle peripheral retinal changes in normal aging^17^. It is also a distance metric and hence likely less variable than traditional retinal oximetry methods^17^.

In this study, we investigate three novel questions in normal aging:1) Does the pCFZ remodel or increase in size?; 2) Beyond group means, do individual differences exist for the perivenule CFZ?; and 3) Is the mean difference in the periarteriole and perivenule CFZ size in cognitively unimpaired older adults significantly different from what was found in cognitively unimpaired younger adults? This current study builds on our previous work characterizing the pCFZ in cognitively unimpaired young adults by characterizing and quantifying the pCFZ as a biomarker of normal aging.

## Methods

### Study participants

20 cognitively unimpaired young adults (mean age: 26 ± 3 years; age range: 22–30 years; 10 males and 10 females) and 20 cognitively unimpaired older adults (mean age: 64 ± 6 years; age range: 55–74 years; 8 males and 12 females) were involved in this current study. All participants had refractive errors of ≤  ± 5.00 DS (spherical equivalent; equivalent axial length of ~ 21–26 mm) to prevent significant differences in retinal magnification in the OCTA images as noted by Bennett’s formula^26^. Characteristics of the cognitively unimpaired young adults have been reported previously^17^. Cognitively unimpaired older adults were recruited at Butler Hospital, Providence, RI, as part of the *Atlas of Retinal Imaging in Alzheimer’s Study* (ARIAS, NCT03862222) and had best corrected visual acuity of at least 20/30 (LogMAR ~ 0.18), Montreal Cognitive Assessment (MoCA) scores of ≥ 26^27,28^, and Repeatable Battery for the Assessment of Neuropsychological Status Update (RBANS-U) Delayed Memory Index (DMI) scores of ≥ 85^29,30^. For all participants, further inclusion criteria involved: 1) absence of systemic diseases such as diabetes, hypertension, cancer or history of cancer; 2) absence of ophthalmic diseases such as diabetic retinopathy, retinal ischemic conditions, glaucoma, and age-related macular degeneration; and 3) absence of usage of retinotoxic drugs such as chloroquine, hydroxychloroquine, and oncologic therapeutics^31^. The estimated sample size of 20 per group (N = 40) was computed with a GPower 3.1 calculator^32^ using the following input parameters; effect size (partial eta squared) of 0.28, α of 0.05, power of 0.80, 3 independent variables (sex, vessel type, distance from fovea), and two levels for sex (male, female) and vessel type (arteriole, venule) as done previously^17^. The study adhered to the tenets of the Declaration of Helsinki, and informed consent was obtained from all subjects. This study is part of the *Atlas of Retinal Imaging in Alzheimer’s Study* (ARIAS; authors P.J.S. & S.S. Co-PI’s) and was approved by the BayCare Institutional Review Board (IRB).

### Image acquisition with OCTA

The OCTA retinal imaging acquisition module used for the cognitively unimpaired young adults has been previously reported^17^. Using OCTA device from the same vendor and similar image acquisition module, the cognitively unimpaired older adults were dilated with one drop of Tropicamide (Myadricyl 1%) per eye prior to imaging. There was a 15-min wait time from dilation to image acquisition. All imaging procedures were completed for both the right and the left eye. We obtained 20 × 20-degree OCTA images consisting of 512 b-scans, 512 A-scans per b-scan, 12 micron spacing between the b-scans and 5 frames averaged per each b-scan location of the central fovea and of paired arterioles and venules with their surrounding capillaries inferior to the fovea (Spectralis HRA + OCT; Eye Explorer version 1.10.4.0; Heidelberg Engineering, Heidelberg, Germany)^31^. The signal quality values of all our OCTA images from the vendor software was at least 20 to ensure good image quality^31^. To ensure independence of sampling, only one eye was selected for the purpose of image analysis based on image signal quality. In cases, where both eyes had similar image signal quality, either eye was randomly selected.

### Processing of OCTA images

**Figure 1:**
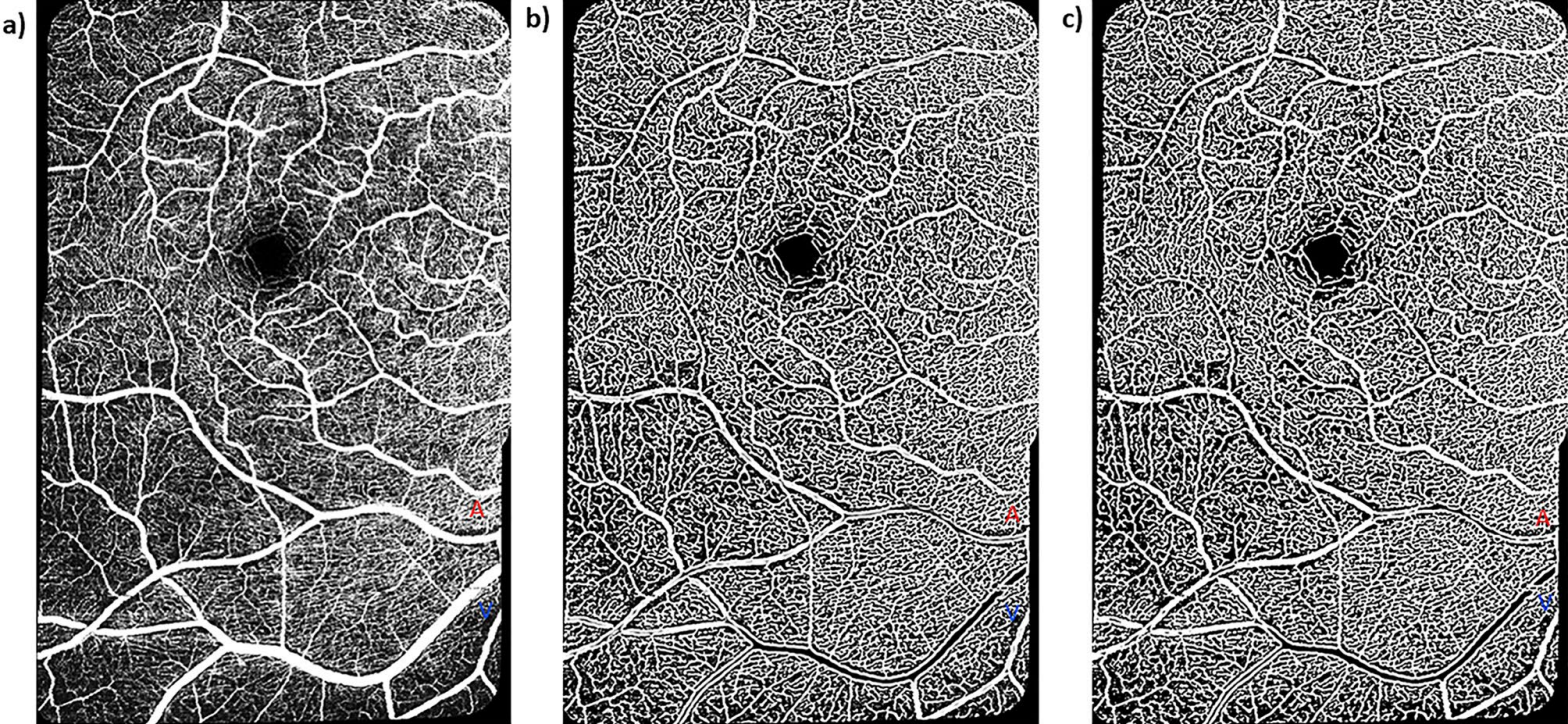
Montaged 20 × 20-degree OCTA images of the central foveal region and paired arterioles and venules in the superficial vascular plexus of a 63-year-old cognitively unimpaired male. (**a**) Raw montaged OCTA image before image processing. (**b**) Vesselness filtered image in Matlab. (**c**) Vesselness filtered and Otsu thresholded image in Matlab. Red “A” and blue “V” represent paired arterioles and venules, respectively. Periarteriole CFZ can be seen dark gaps around the arteriole (Red “A”) while the perivenule CFZ can be seen as dark gaps around the venule (Blue “V”). Periarteriole CFZ can be seen as larger than the perivenule CFZ.

As done previously for the cognitively unimpaired young adults^17^, the superficial vascular plexus (SVP) was the vascular layer of interest for the cognitively unimpaired older adults. The SVP is defined as the composite retinal vasculature from the inner limiting membrane to the inner plexiform layer/inner nuclear layer boundary. The SVP images were then exported as .tiff files into an image editing software (Adobe, Inc) and cropped to include only the OCTA images of interest. Images of the central foveal region and those of paired arterioles and venules inferior to the fovea were then automatically montaged using an image processing software (i2k Retina software) to generate a wider field of view of the paired vessels (Fig. 1a). The montaged images were then also exported into a custom programming software (Matlab, Mathworks). First, a vesselness filter was applied to the images^33^ to increase the probability of resolving a vessel at a specific location in the image when it is actually present versus noise or motion artifact (Fig. 1b). Next, Otsu thresholding method^34^ was applied to the resultant image to reduce background noise (Fig. 1c).

### Computation of Euclidean distances for pCFZ, vessel distance from fovea, vessel diameter, FAZ size, and FAZ effective diameter

**Figure 2:**
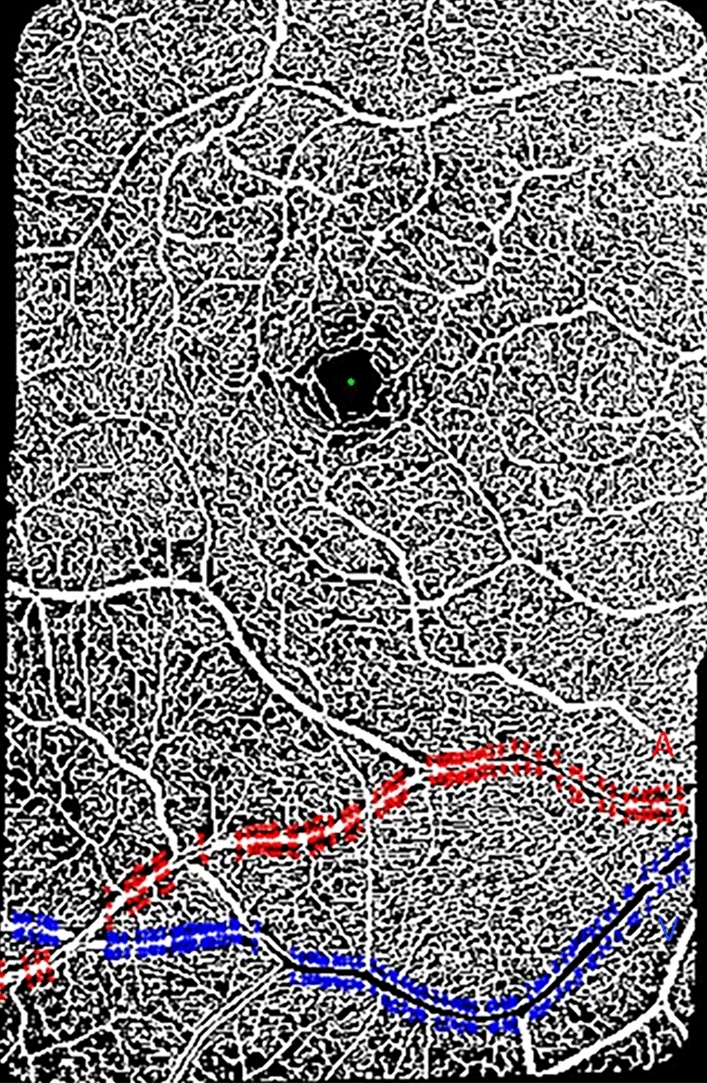
A vesselness filtered and Otsu thresholded montaged 20 × 20-degree OCTA image of the superficial vascular plexus in Matlab of a 63-year-old cognitively unimpaired male. The image shows the central fovea (FAZ area = 0.27 mm^2^; FAZ effective diameter = 586 µm) and a paired arteriole (Red “A”) and venule (Blue “V”), 13.6 and 17.3 degrees, respectively, inferior to the fovea. Evenly sampled points around the arteriole or venule showing the periarteriole (70.2 µm) and perivenule (53.5 µm) CFZs are shown. The arteriole diameter = 96.0 µm and the venule diameter = 129 µm. Evenly sampled points include coordinates from the center of the fovea (green dot), points on the top/bottom edge of an arteriole (red dots) or venule (blue dots), and points from the middle of a capillary superior/inferior to an arteriole (red dots) or venule (blue dots).

Euclidean distances for the pCFZ, vessel distance from fovea, and vessel diameter were computed using similar formulas previously applied for the cognitively unimpaired young adults (Eqs. 1–3)^17^. We computed the pCFZ width in microns as the linear distance from an arteriole (periarteriole CFZ) or venule (perivenule CFZ) to the middle of the nearest capillary. The middle of the nearest capillary was used instead of the edge of the capillary because OCTA does not have enough lateral resolution to truly resolve the edge of a lumen of a capillary compared to other advanced retinal imaging modalities such as AOSLO. A custom Matlab program automatically recorded x and y coordinates from points evenly sampled perpendicular to an arteriole or venule and the middle of the nearest capillary to an Excel file (Fig. 2). The evenly sampled points in Matlab included points perpendicular above and below a paired arteriole or venule as well as the center of the fovea.

Equations (1)–(3) were then used to compute the Euclidean distances for the pCFZ (periarteriole and perivenule CFZ), vessel distance from the fovea, and vessel diameter respectively using the x and y coordinates written by Matlab into Excel. The outcome of each equation produced an Euclidean distance in pixels. For the pCFZ width (Eq. 1) and the vessel diameter (Eq. 3), the Euclidean distances in pixels were then converted into microns by multiplying them by the micron-to-pixel ratio in the x and y directions, as computed from the vendor software fiducial marks (Heidelberg Engineering). For the vessel distance from the fovea (Eq. 2), each Euclidean distance in microns was divided by 300 to convert them into degrees (assuming 300 µm =  ~ 1 degree).

1 $${\text{pCFZ width}} = \left[ {({\text{X}}_{{{\text{large}}\;{\text{vessel}}\;{\text{edge}}}} - {\text{X}}_{{{\text{capillary}}}} )^{2} + ({\text{Y}}_{{{\text{large}}\;{\text{vessel}}\;{\text{edge}}}} - {\text{Y}}_{{{\text{capillary}}}} )^{2} } \right]^{1/2}$$

where large vessel refers to a paired arteriole or venule and capillary represents the middle of the nearest capillary (Fig. 2).

2 $${\text{Vessel}}\;{\text{distance}}\;{\text{from}}\;{\text{fovea}} = \left[ {({\text{X}}_{{{\text{vessel top}}}} - {\text{X}}_{{{\text{fovea}}}} )^{2} + ({\text{Y}}_{{{\text{vessel top}}}} - {\text{Y}}_{{{\text{fovea}}}} )^{2} } \right]^{1/2}$$

where vessel top refers to the top of a paired arteriole or venule and fovea represent the middle of the FAZ (Fig. 2).

3 $${\text{Vessel}}\;{\text{diameter}} = \left[ {({\text{X}}_{{{\text{vessel top}}}} - {\text{X}}_{{{\text{vessel}}\;{\text{bottom}}}} )^{2} + ({\text{Y}}_{{{\text{vessel}}\;{\text{top}}}} - {\text{Y}}_{{{\text{vessel}}\;{\text{bottom}}}} )^{2} } \right]^{1/2}$$

where vessel top/bottom refers to the top/bottom of a paired arteriole or venule (Fig. 2). The area finding tool (lasso tool) of the vendor software was used to delineate and compute the FAZ area in mm^2^. FAZ effective diameter was then computed from the FAZ area values. The FAZ effective diameter in microns was defined as the diameter of a circle whose area was equivalent to the known FAZ areas; FAZ effective diameter = (4*FAZ area/π)^1/2^^17^.

### Statistical analyses

All statistical analyses were done using statistical software (IBM SPSS Statistics for Windows, Version 26; IBM Corp., Armonk, NY, USA) and Microsoft Excel 365 (Microsoft Corp., Redmond, WA, USA) with Real Stats Add-In. All values were presented as mean ± SD. The Shapiro Wilk test of normality was used to test the Gaussian distribution of our data. All outcome variables involved in our data analysis did not significantly differ on this test, with the exception of the FAZ effective diameter of the cognitively unimpaired young participants. We therefore performed the Mann Whitney U test when comparing the FAZ effective diameter between the two groups. For the rest of our data analysis, parametric tests were performed. We previously found the perivenule CFZ to be significantly positively associated with vessel distance from the fovea and vessel diameter in cognitively unimpaired young adults^17^. Hence, vessel distance from the fovea, and vessel diameter served as covariates when comparing the periarteriole and perivenule CFZ between cognitively unimpaired older and younger adults. An independent samples t-test was performed to compare the covariates (Table 1) and FAZ area between the two groups. A one-way analysis of covariance (ANCOVA) was then performed to compare the pCFZs (perivenule and periarteriole CFZ) between the two groups controlling for the specific covariates that came out significant from the t-tests (Table 1). A one-way ANCOVA was performed to compare the pCFZ between the two groups while controlling for vessel diameter (Table 1). Partial eta squared (η^2^_p_) served as our effect size measure from the ANCOVA analysis. A partial eta squared of 0.01 was considered as small, 0.09 considered as medium, and 0.25 considered as large. A paired samples t-test was performed to compare the periarteriole and perivenule CFZ for the cognitively unimpaired older group. We performed a one sample t-test comparing the mean difference in the pCFZ width in the cognitively unimpaired older adults to a set value of 24.5 µm (difference in the pCFZ width in the cognitively unimpaired young adults). Pearson product moment correlation was performed to assess the association between the pCFZ and FAZ parameters in the cognitively unimpaired older adults only. Microsoft Excel 365 (Microsoft Corp., Redmond, WA, USA) with Real Stats Add-In was used to compare the co-efficient of variation (CV) of periarteriole CFZ vs. perivenule CFZ, and pCFZ vs. FAZ parameters in the cognitively unimpaired older adults only. We computed predicted perivenule CFZs and one-tailed 95% CI of the predicted values using the perivenule CFZ regression equation of the cognitively unimpaired young adults [29.1 + 0.571 (venule distance from the foveal center) + 0.037 (venule diameter) + 0.259 (sex)]^17^ to assess if individual differences in the values of the cognitively unimpaired older adults exist rather than just using group means. A *p*-value of < 0.05 was considered statistically significant.

**Table 1:** Vessel distance from the fovea and vessel diameter compared between cognitively unimpaired younger and older adults.

Covariates	Cognitively unimpaired older adults (Mean ± SD)	Cognitively unimpaired young adults (Mean ± SD)	*P*-value
Arteriole diameter	90.7 ± 15.2 µm	102 ± 13.4 µm	0.02*
Venule diameter	118 ± 13.4 µm	129 ± 19.3 µm	0.04*
Arteriole distance from fovea	14.6 ± 1.76 degrees	14.2 ± 3.22 degrees	0.70
Venule distance from fovea	14.9 ± 1.56 degrees	14.6 ± 2.39 degrees	0.54

*Covariates controlled for in the analysis of covariance (ANCOVA) comparing the pCFZs between the cognitively unimpaired older and young adults.

## Results

### Characteristics of pCFZ in cognitively unimpaired older adults

**Figure 3:**
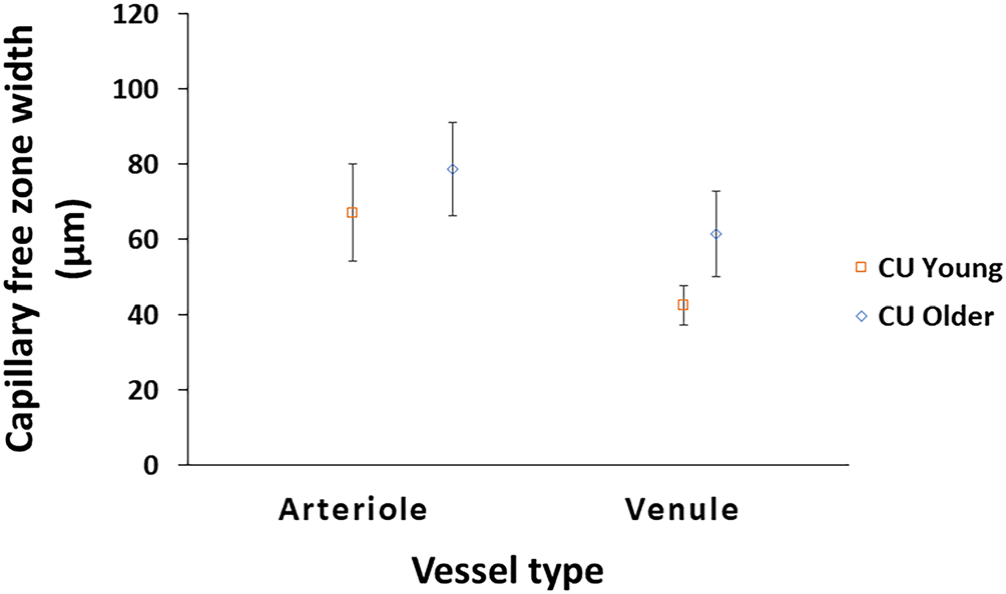
Mean capillary free zone (CFZ) width with standard deviation error bars for arterioles and venules in cognitively unimpaired (CU) young and older adults. The periarteriole CFZ width is significantly greater than the perivenule CFZ width in CU older adults (*p* < 0.001) similar to that reported previously^17^. The periarteriole CFZ width of the CU older adults is significantly greater than that of the CU young adults (*p* = 0.034). The perivenule CFZ width of the CU older adults is also significantly greater than that of the CU young adults (*p* < 0.001).

The periarteriole CFZ width (78.8 ± 12.3 µm) was significantly larger than the perivenule CFZ width (61.5 ± 11.3 µm), t(19) = 10.1, *p* < 0.001 in the cognitively unimpaired older adults (Fig. 3). The mean difference between the periarteriole and perivenule CFZ width (17.3 µm) was significantly less than that found in the cognitively unimpaired young adults (24.5 µm), t(19) = − 4.16, *p* = 0.001, indicating that the perivenule CFZ remodeled to a greater extent, on average, in normal aging compared to the periarteriole CFZ. The CV of the periarteriole CFZ (0.16) did not significantly differ from that of the perivenule CFZ (0.18), z = − 0.69, *p* = 0.49.

### Comparison of the pCFZ and FAZ parameters between cognitively unimpaired older and young adults

**Figure 4:**
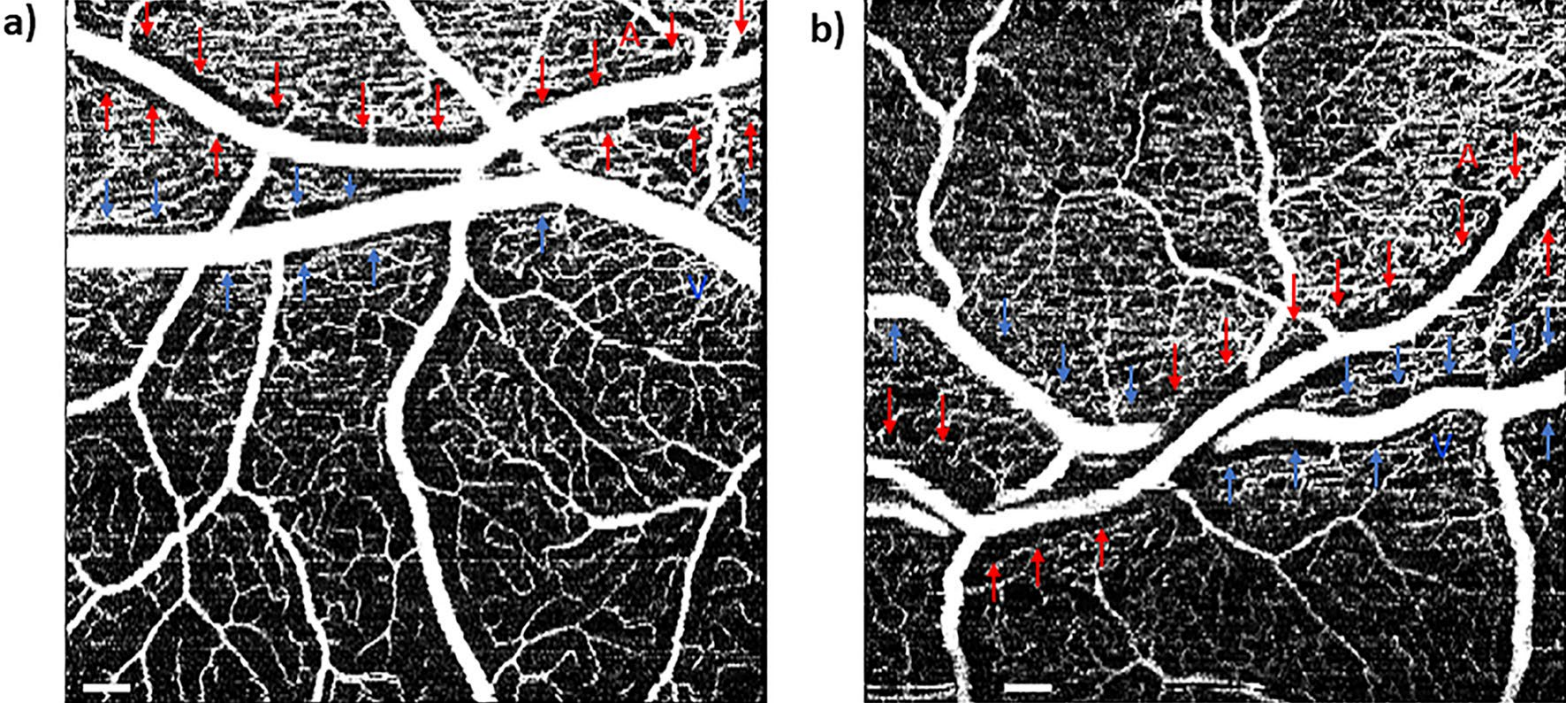
Raw OCTA images showing an example of the remodeling of the pCFZs in normal aging for 10 × 10-degree region of the retina. 10 × 10-degree images are shown here to demonstrate detailed remodeling of the pCFZ in normal aging. (**a**) OCTA image of a 23-year-old cognitively unimpaired male showing paired arteriole (red “A”) and venule (blue “V”) at 15.8 and 17.4 degrees, respectively, inferior from the fovea in the superficial vascular plexus (SVP). Periarteriole and perivenule CFZs are indicated by red and blue arrows, respectively. Periarteriole CFZ (68.6 µm) can be observed as larger than the perivenule CFZ (44.7 µm). (**b**) OCTA image of the SVP of a 62-year-old cognitively unimpaired female showing the peripheral periarteriole (red “A” and arrows) and perivenule (blue “V” and arrows) CFZs, 14.6 and 14.2 degrees, respectively, inferior to the fovea. The periarteriole (97 µm) and perivenule (64.9 µm) CFZs can be observed as larger than those observed in the cognitively unimpaired young participant. Scale bar: 200 µm.

**Figure 5:**
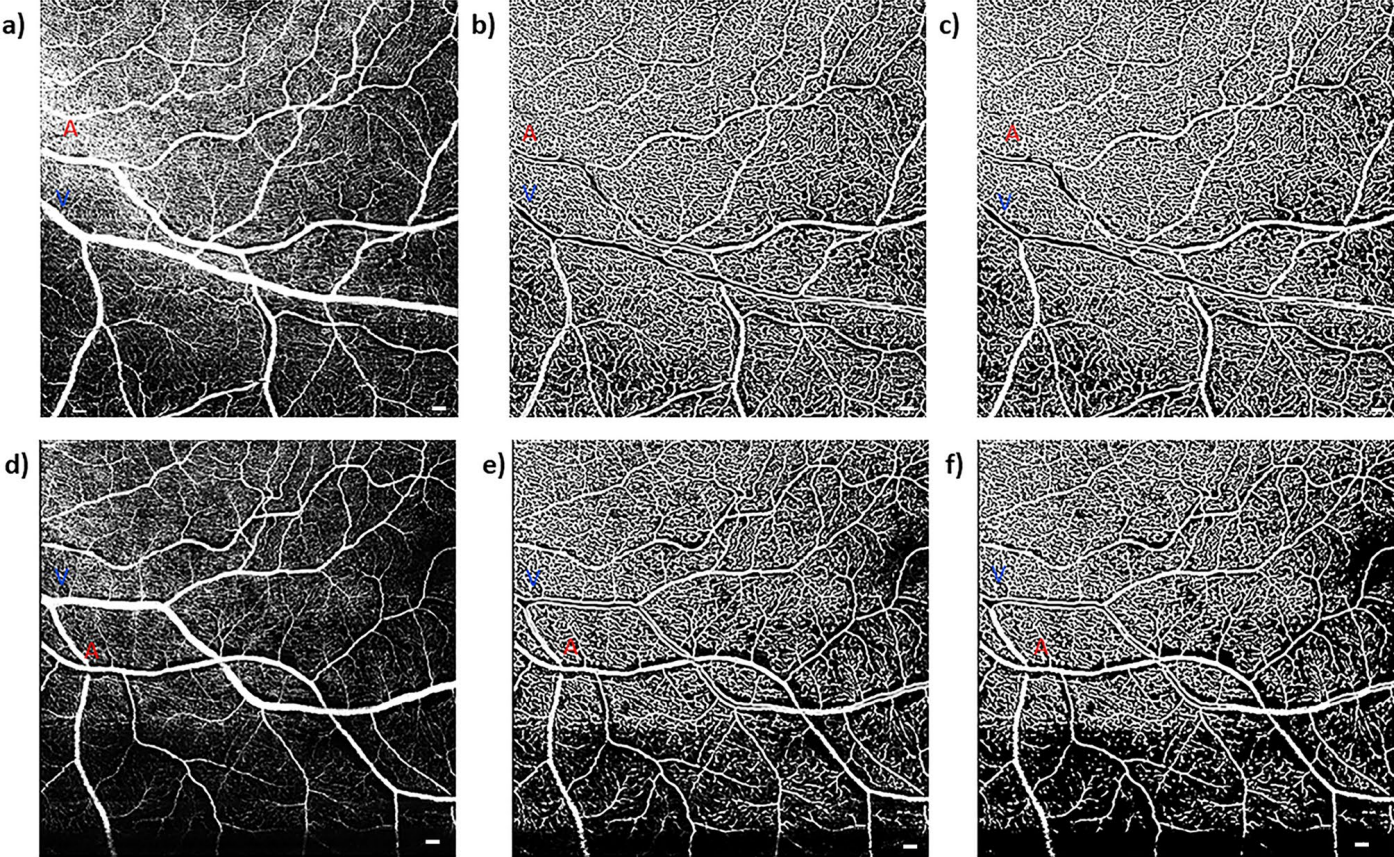
Raw and processed OCTA images showing a sex-matched example of the remodeling of the pCFZ in normal aging for a 20 × 20-degree region of the retina. (**a**) and (**d**) represent raw OCTA images of a 28 and 66-year-old cognitively unimpaired female, respectively. (**b**) and (**e**) represent corresponding vesselness filtered images in Matlab. (**c**) and (**f**) represent corresponding vesselness filtered and Otsu thresholded images in Matlab. Red “A” and blue “V” represent paired arterioles and venules, respectively. The dark gaps around the arteriole (periarteriole CFZ; 111 µm; vessel distance from fovea = 14.2 degrees) and venule (perivenule CFZ; 93.2 µm; vessel distance from fovea = 13.9 degrees) of the cognitively unimpaired older adult can be observed as larger than that around the arteriole (periarteriole CFZ; 73.4 µm; vessel distance from fovea = 13.8 degrees) and venule (perivenule CFZ; 57.4 µm; vessel distance from fovea = 16.1 degrees) of the young participant. Scale bar: 200 µm.

The ANCOVA showed that the mean periarteriole CFZ width in the cognitively unimpaired older adults (78.8 ± 12.3 µm) was significantly larger than that of the cognitively unimpaired young adults (67.2 ± 12.8 µm), F_(1,37)_ = 4.87, *p* = 0.034, with a medium effect size (η^2^_p_) = 0.12, while controlling for arteriole diameter as a covariate (Figs. 3, 4, 5). The mean periarteriole CFZ of the cognitively unimpaired older adults was also outside the one-tailed 95% CI of the cognitively unimpaired young adults (68.6 μm). Similarly, the mean perivenule CFZ width in the cognitively unimpaired older adults (61.5 ± 11.3 µm) was significantly greater than that of the cognitively unimpaired young adults (42.7 ± 5.17 µm), F_(1,37)_ = 46.9, *p* < 0.001, with a large effect size (η^2^_p_) = 0.56, while controlling for venule diameter as a covariate (Figs. 3, 4, 5). The mean perivenule CFZ of the older group was outside the one-tailed 95% CI of the younger group (43.5 μm).

Beyond group means, each individual perivenule CFZ width of the older adults was greater than the predicted values and one-tailed 95% CI of the predicted values of younger adults with similar vessel parameters, except for two participants (S003 and S010; Table 2) whose actual computed values were greater than the predicted values but within the 95% CI. Figures 4 and 5 show examples of the remodeling of the pCFZs between younger and older adults for raw and processed images, respectively. Raw OCTA images are shown in Fig. 4 to demonstrate that even prior to customized processing of the images in Matlab, remodeling of the pCFZs in normal aging can be visually demonstrated. Customized processing of the images in Matlab as shown in Fig. 5 visually elucidates further the remodeling of the pCFZ in normal aging.

**Table 2:** Actual computed perivenule CFZ width of all cognitively unimpaired (CU) older participants as well as predicted perivenule CFZ and one-tailed 95% CI of the predicted values of younger adults with similar vessel parameters are compared.

CU older participants	Perivenule CFZ width of CU older adults (µm)	Predicted perivenule CFZ width of CU young adults (µm)	One-tailed 95% CI for predicted perivenule CFZ width of CU young adults (µm)
S001	68.2	41.8	53.4
S002	49.5	42.6	48.7
S003*	46.9	41.2	47.0
S004	56.5	41.2	49.0
S005	58.2	40.1	48.3
S006	61.4	43.1	52.4
S007	53.4	44.0	51.0
S008	55.1	42.6	50.0
S009	64.9	42.1	52.4
S010*	47.7	42.2	48.0
S011	63.8	41.5	51.5
S012	54.5	42.2	49.4
S013	93.2	41.9	62.6
S014	75.4	44.2	58.3
S015	77.3	44.9	59.6
S016	66.8	42.7	53.7
S017	55.9	43.2	50.8
S018	52.7	41.8	48.6
S019	67.1	42.7	53.8
S020	60.8	42.4	51.5

All the actual computed perivenule CFZs are greater than the predicted and one-tailed 95% CI of the predicted values except for two study participants.

*Actual computed values are greater than the predicted values but within the 95% CI of the predicted values.

The FAZ area of the cognitively unimpaired older adults (0.34 ± 0.127 mm^2^) did not differ significantly from that of the cognitively unimpaired young adults (0.368 ± 0.114 mm^2^), t(38) = 0.73, *p* = 0.47. Similarly, the FAZ effective diameter of the cognitively unimpaired older adults (647 ± 123 µm, median = 649 µm) did not differ significantly from that of the cognitively unimpaired young adults (674 ± 120 µm, median = 700 µm), *U* = 148, *p* = 0.16.

### Relationship between the pCFZ and FAZ parameters in cognitively unimpaired older adults

There was no significant association between the periarteriole and perivenule CFZ width and FAZ area (r = 0.08, *p* = 0.75; r = 0.23, *p* = 0.34, respectively). Similarly, there was no significant association between the periarteriole and perivenule CFZ width and FAZ effective diameter (r = 0.10, *p* = 0.66; r = 0.22, *p* = 0.36, respectively). The CV of the FAZ area (0.37) was significantly greater than that of the periarteriole (0.16; z = 3.35, *p* = 0.001) and perivenule CFZ (0.18; z = 2.76, *p* = 0.006). Interestingly, the CV of the FAZ effective diameter (0.19) did not significantly differ from that of the periarteriole (0.16; z = 0.84, *p* = 0.40) and the perivenule CFZ (0.18; z = 0.16, *p* = 0.88).

## Discussion

Using OCTA retinal imaging device and software from the same vendor (Spectralis HRA + OCT, Heidelberg Engineering, Heidelberg, Germany) and similar imaging acquisition protocol for our cognitively unimpaired young and older participants, we characterized and quantified the remodeling of the pCFZs in normal aging. We found the periarteriole CFZ to be significantly larger than the perivenule CFZ in the cognitively unimpaired older adults, consistent with our previous results in the cognitively unimpaired young adults^17^. The difference in the width of the pCFZs in the cognitively unimpaired older adults was significantly smaller than that of the cognitively unimpaired young adults indicating a greater remodeling of the perivenule CFZ in normal aging compared to the periarteriole CFZ. The pCFZs in the older adults was significantly larger than that of the young adults with moderate and large effect sizes respectively for the periarteriole and perivenule CFZs and beyond group means, there were individual differences when 1:1 comparisons were made between the older and younger participants for the perivenule pCFZs, indicating potential clinical usability of this metric to detect age-related retinal vascular changes. FAZ parameters did not significantly differ between the two groups and the CV of the FAZ area was significantly greater than that of the periarteriole and perivenule CFZ in the older group. Interestingly, the CV of the FAZ effective diameter did not significantly differ from that of the periarteriole and perivenule CFZ in the older group indicating a more favorable usability of this metric.

The retinal neurovascular unit consists of blood vessel endothelial cells, pericytes, neurons, astrocytes, and Müller cells that are intimately connected to form the inner retinal blood barrier^35^. The inner retinal blood barrier essentially controls nutrient flow to the neural retinal; specifically, the inner retinal neurons^35^. A pCFZ represents the distance that oxygen and nutrient have to diffuse to reach the neural retina, and therefore serves as a metric of retinal tissue oxygenation.^17^ The pCFZs are formed based on the concept of oxygen saturation in the retinal arterioles and venules; essentially larger periarteriole CFZs are expected compared to perivenule CFZs since retinal arterioles contain large concentration of oxygenated blood compared to venules and hence a reduced need for capillaries to exist closely to the arterioles and vice versa^17,21–25^. Our results of periarteriole CFZs being larger than the perivenule CFZs in cognitively unimpaired older adults is consistent with our hypothesis and similar to previous findings^17,23,25^. Interestingly, the difference in the width of the pCFZs in the older adult group was significantly smaller than that of the younger adult group, indirectly indicating that even though there is greater oxygen saturation in both the arterioles and venules in older adults, the magnitude of increase in oxygen saturation for the venules in older adults was greater compared to the arterioles.

In normal aging, there is reduced vessel density^4,14^, and atrophy of the inner retinal neurons^9–11^ leading to a paradoxical increased oxygen saturation (reduced oxygen extraction) in the arterioles and venules^12,13^. Since the pCFZs are formed to regulate oxygen diffusion to meet requirements of the surrounding neuropil^17,21,23^, it follows that the higher the oxygen saturation in the arterioles and venules (reduced need for capillaries to exist closer to the retinal vessels), the wider the pCFZ. We found larger pCFZs in the older group compared to the young group, indicating reduced oxygen uptake in older adults compared to younger adults. The group difference effect size for the perivenule CFZ was large while that of the periarteriole CFZ was moderate, indicating the potential use of the pCFZ width as a biomarker for age-related retinal vascular unit changes. Beyond group means, there were individual differences for the perivenule CFZs indicating sensitivity of the pCFZ as a biomarker of normal age-related retinal vascular changes and corroborating our previous results which found the perivenule CFZ to be more consistent compared to the periarteriole CFZ^17^.

The FAZ area as a clinical biomarker for normal age and disease associated retinal vascular changes has various limitations^15–17^, the computation of retinal vessel density can be affected by noise in the image along with variable anatomical features such as vessel diameter^20^, and traditional retinal oximetry methods are highly variable^18,19^. The FAZ consists of a single layer of lacy capillaries and as a metric of size, it may saturate with no further increase in size even with increasing disease severity^17^. It is also highly variable^15–17^ and the capillaries of the FAZ are limited to deeper retinal layers where there is little influence of the RNFL and RGCs which are known to atrophy in normal aging^17^. Our results showed no significant difference in FAZ area or effective diameter between the two groups, consistent with previous results that did not demonstrate remodeling of the FAZ area in normal aging^6–8^. The above expatiation of the limitations of the FAZ area may have accounted for the lack of significant difference between the groups. However, we have to also consider the limited sample size of our data as a possible explanation. Interestingly, the CV of the FAZ area was significantly larger than that of the pCFZs while that of the effective diameter did not significantly differ from the pCFZ. This indicates that the FAZ effective diameter may be a more useful clinical retinal vascular metric to detect age-related retinal vascular changes in the retina compared to the FAZ area. The above findings of the FAZ metrics indicate that the pCFZ may be more sensitive to age-related changes in the retinal neurovascular unit than the FAZ.

The pCFZ has a potential clinical application as a retinal vascular biomarker for early detection of Alzheimer’s disease (AD)^36–40^. The human retina is an extension of the brain as both are derived from the neural ectoderm, both are supported by glia and astrocytes, and both are metabolically demanding. Also, RGCs are similar to cerebral cortex neurons, and cerebral small vessels are similar to retinal vessels^41^. It is well established that AD is not limited to the brain but also affects the retina^36–40^. The neurodegeneration in the brain of AD patients is associated with reduced RNFL and RGC layer thickness^40,42^. The human retina is therefore ideally positioned as a non-invasive window to the brain for early AD risk detection and disease monitoring. In AD, there is increased thinning of the RGCs and RNFL compared to normal aging, concomitant with the atrophy of the frontal cortex and hippocampus^40,42–44^. As a result, a higher oxygen saturation in the retinal arterioles and venules^45,46^ and a corresponding larger peripheral CFZ in the AD retina would be expected compared to cognitively unimpaired older adults. Such an investigation should also include measures of visual sensory metrics such as field of vision and sensitivity to contrast. 24–2 and 30–2 protocols can be done for the field of vision assessment in order to target the peripheral retina. The above analogy represents the next line of steps in terms of future research for our current research lab.

We recommend montages of OCTA images for the computation of pCFZ width while using retinal imaging device from the same vendor across groups of participants. In our current study, OCTA retinal imaging device from the same vendor (Spectralis HRA + OCT, Heidelberg Engineering, Heidelberg, Germany) was used to image both groups of participants. Comparing retinal vascular metrics from OCTA devices from different vendors at this point in the retinal imaging field is discouraged^31^. Montages of OCTA images can include 10 × 10-degree (3 mm × 3 mm; for our younger participants published previously)^17^ and 20 × 20-degree (6 mm × 6 mm) images from the same vendor software. pCFZs computed from 10 × 10-degree and 20 × 20-degree OCTA montages taken with the same vendor software can be compared so far as the following conditions are met; (1) Using the micron to pixel ratio in the x and y directions from the vendor software fiducial marks for each individual image, and (2) Using the same defined boundary for the retinal vascular plexus of interest (SVP for example in this current study). Even with periodic software updates for some vendors (Heidelberg Engineering for e.g.), there is always flexibility to customize the boundaries of the retinal vascular plexus of interest to be the same as previous. The size of the FAZ area for example will not differ in a 10 × 10-degree image compared to a 20 × 20-degree if the same vendor software is used and the above conditions are met. Measures of vessel density will differ between the two because the areas are different. It is imperative to mention that a 10 × 10-degree montage (depending on the number of single images used in the montage) generates a field of view of the OCTA image that is greater than 10 degrees and similar to what can be achieved in a 20 × 20-degree montage. So far as (1) the same vendor software is used, (2) micron to pixel ratio in the x and y directions from the fiducial marks for each individual image is used, and (3) the same retinal boundary definition is used for the vascular plexus of interest, covariates of vessel distance from the fovea, and vessel diameter^17^ are what need to be controlled for when comparing pCFZs across of groups of pariticpants. However, when possible, similar field of view should be used in OCTA montages for each group of participants for the computation of pCFZ width so a constant fiducial mark from the vendor software can be applied to the images.

Limitations of the results of our current study are embedded in the limits of lateral resolution of OCTA technology as a whole, limited age-range for our young participants, and future covariates/associations that need to be investigated. OCTA however has better axial resolution than other superior lateral resolution devices such as AOSLO. Tighter CI of the pCFZs of the young participants were also provided despite the limited age range. Other covariates/associations such as sex^47^, linear distance traversed for a vessel especially for montaged images and associations with retinal thickness and other visual sensory measures like sensitivity to contrast and field of vision in a diseased group all need to be explored in future studies.

In summary, we characterized and quantified the remodeling of the pCFZs in normal aging. We showed that the perivenule CFZ remodeled to a greater extent compared to the periarteriole CFZ in normal aging further corroborating the consistency of the perivenule CFZ. There were large and moderate effects sizes for the group differences in the pCFZ width indicating the potential use of the pCFZ width as a biomarker for age-related retinal vascular unit changes. Beyond group means, there were individual differences for the perivenule CFZ in the older group indicating sensitivity of this metric as a retinal vascular measure for normal aging. We showed high variability of the FAZ area compared to the pCFZ width and indicated that the FAZ effective diameter may be a more useful retinal vascular metric than FAZ area. The FAZ metrics did not significantly differ between the cognitively unimpaired young and older group. The findings of the FAZ metrics indicate that the pCFZ may be more sensitive to age-related retinal vascular changes than the FAZ. Beyond showing the remodeling of the pCFZ in normal aging, the next steps of research would include demonstrating its applicability in AD and cerebral amyloid angiopathy^48^.

## Data Availability

Data used in this current study are available from the corresponding author upon appropriate data sharing agreement with University of Rhode Island (URI) and BayCare Health.
